# Empowering Support for Family Members of Brain Injury Patients in the Acute Phase of Hospital Care: A Mixed-Methods Systematic Review

**DOI:** 10.1177/10748407231171933

**Published:** 2023-05-16

**Authors:** Julia Lindlöf, Hannele Turunen, Tarja Välimäki, Justiina Huhtakangas, Sofie Verhaeghe, Kirsi Coco

**Affiliations:** 1 University of Eastern Finland, Kuopio, Finland; 2 University of Helsinki, Finland; 3 Ghent University, Ghent, Belgium

**Keywords:** empowering support, traumatic brain injury, family members, acute phase of hospital care, mixed-methods systematic review

## Abstract

This review aimed to identify and synthesize empowering support for the family members of patients in the acute phase of traumatic brain injury hospital treatment. CINAHL, PubMed, Scopus, and Medic databases were searched from 2010 to 2021. Twenty studies met the inclusion criteria. Each article was critically appraised using the Joanna Briggs Institute Critical Appraisals Tools. Following a thematic analysis, four main themes were identified about the process of empowering traumatic brain injury patients’ family members in the acute phases of hospital care: (a) needs-based informational, (b) participatory, (c) competent and interprofessional, and (d) community support. This review of findings may be utilized in future studies focusing on designing, implementing, and evaluating an empowerment support model for the traumatic brain injury patient’s family members in the acute care hospitalization to strengthen the current knowledge and develop nursing practices.

Traumatic brain injury (TBI) is functional or structural damage to the brain caused by a sudden external injury. TBI can be classified as a mild, moderate, or severe brain injury. Moderate and severe brain injuries in the acute phase often require hospital treatment (Capizzi et al., 2020). Approximately 5.3 million people in the United States and 7.7 million people in the European Union (G. Liu et al., 2021) suffer from various symptoms and problems caused by a TBI, including impaired attention, difficulty with memory, depression, impulsivity, poor decision-making, aggressive behavior, slowness, fatigue, and mental disorders (Capizzi et al., 2020; Rasmussen et al., 2020). A considerable number of people with brain injuries are below 25 years old, although brain injuries have also increased among older people (Nguyen et al., 2016). After hospital discharge, family members (FMs) are often the primary caregivers for a TBI survivor, offering daily support and executing demanding care procedures (McIntyre et al., 2020). FMs must adapt to this new, unexpected role (McIntyre et al., 2020), and as a result, they often experience difficulties managing the TBI survivor care process (Kivunja et al., 2018), and need empowering support (Sakanashi & Fujita, 2017). Based on the literature, TBIs are a global health problem (Maas et al., 2017), and the number of brain injuries is constantly increasing (Jochems et al., 2021). Therefore, it can be assumed that the number of FMs and caregivers will also increase in the future.

## Background

Empowering the FMs of TBI patients has received little attention in nursing science. Most previous studies have focused on the needs of FMs (de Goumoëns et al., 2018; Kreutzer et al., 2018) and the relationships between life satisfaction (Manskow et al., 2017), perceived burden (Doser & Norup, 2016), and the functioning of the TBI patient and FMs after hospitalization. These studies reported FM’s unfulfilled needs in the acute phase of TBI patient care were related to insufficient emotional support, professional support, and involvement with care (de Goumoëns et al., 2018). In addition, research has reported that FMs’ needs do not decrease over time but actually increase (Anke et al., 2020; de Goumoëns et al., 2018). Furthermore, FMs’ feelings of burden (Doser & Norup, 2016) and depression increased and were related to decreased life satisfaction (Manskow et al., 2017), especially in the context of severe brain injury (Rasmussen et al., 2020). Therefore, professionals should recognize and attend to the needs of FMs in the acute phases of TBI to better support and empower FMs in order to prevent these negative consequences for the individual and the family.

Empowerment is a mutual process multidimensional concept that has been defined in several disciplines, including education, politics (Mehta & Sharma, 2014), social sciences (Rubin & Babbie, 2016), psychology (Jones et al., 2011), feminist studies (Rodwell, 1996), and nursing science (Friend & Sieloff, 2018; Wåhlin, 2017). In nursing science, the concept of empowerment has been studied from the perspectives of patients (Ania-Gonzalez et al., 2022), health care professionals (Papathanasiou et al., 2014), and management (Garcia-Sierra & Fernandez-Castro, 2018), but less from the viewpoint of TBI patients and their FMs.

Empowerment has been described both as a process and an outcome (Friend & Sieloff, 2018). Empowerment as a process means offering hope and confidence and encouraging people to promote their well-being, decision-making, and self-management (Chen & Li, 2009). Empowerment as an outcome, in turn, means that the individual feels able to manage and control their situation (Sakanashi & Fujita, 2017). From an empowerment perspective, FMs require support, knowledge, and guidance from the health care professionals during the acute phase of TBI patient hospital care (Sakanashi & Fujita, 2017) to manage the complex, life-changing situation and adapt to it (Kreutzer et al., 2018). Empowerment of FMs requires that the information a health care professional provides is multifaceted and corresponds to the FM’s expectations and needs in a manner that can also benefit decision-making (Sigurdardottir et al., 2015). Qualities such as authenticity, communication, listening, and equality are needed for an empowered mutual relationship between families and health care professionals, with acceptance and support being the key factors thereby creating an atmosphere where FMs can express their feelings and concerns (Wåhlin, 2017).

The key elements of providing empowering support to FMs relate to equal and trustful relationships between the professionals and the FMs (Sakanashi & Fujita, 2017). FMs can develop a positive belief in themselves and the future in this process. Professional competence to support FMs in achieving the skills needed to manage TBI survivors’ care independently after hospitalization and to overcome challenges through guidance and emotional support are also important in the empowerment process. Furthermore, health care professionals must meet FM’s needs and expectations with the knowledge to reach potential empowerment (Funnell et al., 1991; Nygårdh et al., 2012; Wåhlin, 2017).

Previous systematic reviews have examined the experiences, requests for support, and needs of FMs of TBI patients in the hospital (Coco et al., 2011; Oyesanya, 2017; Wetzig & Mitchell, 2017). According to recent studies (de Goumoëns et al., 2018; Doser & Norup, 2016; Manskow et al., 2017), FMs reported that they did not receive enough information, support, and guidance from health care professionals. As a result, FMs experienced a long-term feeling of burden and a reduced quality of life. There is a gap in the available knowledge from the perspective of providing empowering support for FMs in the acute phase of TBI patients’ hospital treatment. Moreover, there is a lack of nursing recommendations and structured care procedures prepared to support FMs in the acute phase of TBI patients’ hospital treatment.

Research focusing on empowering support for FMs in the acute phase of TBI patient care is significant, both for increasing health care professionals’ awareness of FMs’ needs and for improving care procedures to support and empower FMs experiencing the TBI of a loved one.

This systematic review aimed to identify, critically evaluate, and synthesize available evidence of empowering support for FMs in the acute phase of TBI patient hospital treatment, including emergency care, intensive care unit (ICU) care, and inpatient care. Specifically, we wanted to (a) identify factors that contribute to FMs’ empowerment and (b) understand the empowering support from the perspective of FMs of TBI patients. The research question that guided this study was: What is empowering support for the FMs of TBI patients in the acute phase of TBI patient hospital treatment, and what are the influencing factors?

## Method

### Design

This mixed-methods systematic review explored FMs’ perspective of empowering support in the acute phase of TBI patients’ hospitalization. A convergent integrated design approach was chosen because it enables gathering information about the care procedures that families found helpful and also explored the experiences of FMs to better understand these multifaceted phenomena (Grant & Booth, 2009; Lizarondo et al., 2020). The population, intervention, control, and outcomes format was used for framing the research question (Aslam & Emmanuel, 2010). The literature review was conducted and reported using the Preferred Reporting Items for Systematic reviews and Meta-Analyses (PRISMA) statement (Page et al., 2021) (see Online Supplementary File 1).

### Search Methods

We performed the systematic data retrieval by dividing the research question into thematic entities to define key concepts and construe search terms. We conducted searches in the CINAHL, PubMed, Scopus, and Medic databases; the process also included testing and combining Medical Subject Headings terms. The search strategy with phrases variations is provided in Table 1.

**Table 1.** Search Strategies for the Systematic Mixed Methods Review.

Database	Search options	Items found
CINAHL		
((tbi OR “traumatic brain injur*” OR “brain injury” OR “acquired brain injury” OR “brain injuries” OR “brain injuries traumatic”)) AND ((support* OR guidance OR assist* OR empower*)) AND Need* AND ((“family members” OR spouse* OR husband* OR wife OR relative* OR carers OR caregiver* OR “next of kin” OR family))	Limiters -Peer Reviewed; English Language; Published Date: 2010-2021	409
PubMed		
(“family members” OR spouse* OR husband* OR relative* OR carers OR caregiver* OR “next of kin” OR family) AND (support* OR guidance OR assist* OR empower* OR “empowerment [MeSH]”) AND need* AND (tbi OR “traumatic brain injur*” OR “brain injury” OR “acquired brain injury” OR “brain injuries” OR “brain injuries, traumatic” [MeSH]”)	Filters—from 2010-2021, English, Adult: 19+ years	434
Scopus		
(TITLE-ABS-KEY (“family members” OR spouse* OR husband* OR relative* OR carers OR caregiver* OR “next of kin” OR family) AND TITLE-ABS-KEY ( support* OR guidance OR assist* OR empower*) AND TITLE-ABS-KEY (need* ) AND TITLE-ABS-KEY ( tbi OR “traumatic brain injur*” OR “brain injury” OR “acquired brain injury” OR “brain injury, traumatic”)) AND	(LIMIT-TO (DOCTYPE, “ar”) OR LIMIT- TO (DOCTYPE, “re”)) AND (LIMIT- TO (LANGUAGE, “English”) AND (LIMIT-TO (PUBYEAR, 2010-2021))	654
Medic		
“Family members” spouse* husband* relative* carers caregiver* “next of kin” family AND support* guidance assist* empower* need* AND tbi “traumatic brain injur*” “brain injury” “acquired brain injury” “brain injuries” “brain injuries, traumatic” 2010	Limiters—2010–2021	3

An information specialist’s expertise was used to improve the data set coverage and reliability in the data retrieval process. Inclusion criteria were studies involving adults over 18 years old; the patient’s and FMs’ experiences of TBI; and needs of FMs for support during the acute phase of treatment. In addition, the health care professional’s supportive approaches, nursing practices, and nursing interventions from the perspective of FMs’ empowerment were also examined. Furthermore, factors related to empowering TBI patients’ FMs in the acute phases of hospital treatment were included. In order to obtain a more comprehensive synthesis, numerous qualitative, quantitative, RCT, and mixed methods studies were screened. Exclusion criteria included non-traumatic brain injuries, FMs’ experiences and needs of children with TBI literature reviews, medical intervention, rehabilitation, and outpatient care. Data retrieval was limited to peer-reviewed research articles in English. We did not include gray literature in the data retrieval process. The time period included in all database searches was 12 years (2010–2021). Table 2 presents the criteria for inclusion and exclusion of studies in the review.

**Table 2.** Inclusion and Exclusion Criteria of the Search Strategy.

PICO	Inclusion criteria	Exclusion criteria
Population	Adult TBI patient’s family members (18 years and older)	Children TBI (below 18 years) and their family members Non-traumatic brain injury
Intervention	Policies/practices/nursing interventions provided by healthcare professionals to support/promote empowerment of TBI patient’s family members in the hospital The policy/nursing intervention is carried out in a hospital, in the acute phases of TBI treatment (from the emergency department to the hospital ward)	Non-hospital policies /practices/interventions Policies/practices/interventions focusing on other times than the acute phases of TBI treatment Medical intervention such as clinical trial
Control	A control group is not required	
Outcomes	Factors related to empowerment of the TBI patient’s family members in the acute phases of TBI treatment	Factors related to empowerment after the acute phase of TBI treatment, such as during rehabilitation or at home

*Note.* PICO = population, intervention, control, and outcomes; TBI = traumatic brain injury.

### Study Selection and Data Extraction

The literature selection process proceeded in two phases. The first author (JL) independently carried out report retrieval for the study. In the first phase, duplicates and records marked as ineligible by the automation tool were removed. According to the inclusion and exclusion criteria, two researchers (JL and KC) independently selected studies based on the title and the abstract. Covidence program was used (Kellermeyer et al., 2018) for data extraction. The second phase included reading each study and re-checking whether the study answered the research question and fulfilled the inclusion criteria. Any possible disagreements were discussed with the other members of the research group (TV and HT) to reach a consensus and make the decisions.

## Data Analysis

A thematic analysis was used to analyze and synthesize the findings; the review included qualitative, quantitative, and mixed methods designs. The studies were read, familiarized, and coded by forming a narrative interpretation of the quantitative results (Lizarondo et al., 2020; Vaismoradi et al., 2013). Each publication was analyzed to find expressions describing the FMs’ experiences of receiving empowering support in the acute phase of TBI patient’s hospital treatment. Some of these expressions offered by FMs were narratives (e.g., “*. . .need for continuity of care. . .so it has all been taken care of and then you can free your time to go to work”*), and some were phrases (e.g., “*to receive concrete information on the brain injury and its effect on the future at an early stage”*), and some were single words (e.g., “*. . .empowerment processes. . .*”). These meaningful expressions formed a basis for data reduction, categorization, and abstraction. After this, similar reduced expressions were grouped into categories by comparing their similarities and differences. Categories with similar content were grouped as a subtheme with a name that described the content (e.g., *information about TBI patients’ health conditions in the acute phase*). The subthemes were then grouped into higher-level categories and main themes (e.g., informational support to empower the FMs) (Elo et al., 2014); (see Online Supplementary File 2).

Two reviewers (JL and KC) independently appraised the methodological quality of the studies and performed the quality assessment using Joanna Briggs Institute (JBI) Critical Appraisals Tools: (a) Checklist for Qualitative Research and (b) Checklist for Analytical Cross-Sectional Studies. The JBI critical appraisal checklist includes 10 criteria for qualitative studies and 8 criteria for quantitative studies, addressing the risk of bias in its design, conduct, and analysis (Moola et al., 2020). For each study, two reviewers completed the appraisal step (each reviewer rated each study “Yes,” “No,” “Unclear,” or “Not applicable”). In my opinion, this sentence can be removed here, as it will come up in the next section “Description of Included Studies”. The studies’ strengths were related to a clear description of the research methodology, data collection methods, and data analysis. The inclusion and exclusion criteria of the sample were also clearly described, including the study’s subjects and settings. Weaknesses in reviewed studies related to the lack of description of potential confounding factors and strategies to control them. In total, the selected studies (*N* = 20) were generally of good quality and were not excluded based on their quality assessment.

## Results

### Study Selection

**Figure 1.**
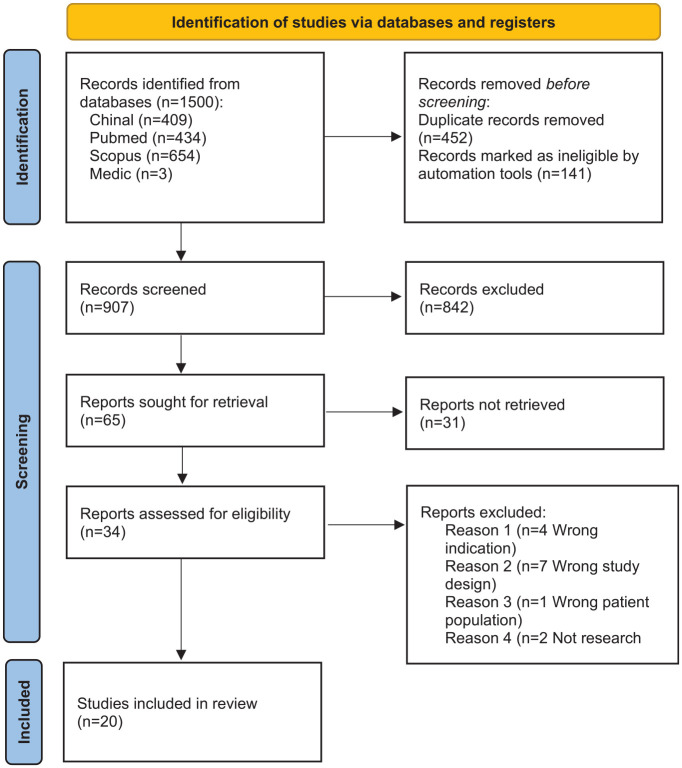
PRISMA Flowchart of the Selection of Included Articles (Page et al., 2021). *Note.* PRISMA = Preferred Reporting Items for Systematic Reviews and Meta-Analyses.

At the first stage of the data retrieval process, the number of hits within the search limits was 1500. After removing duplicates and records marked as ineligible, 907 articles remained. Of these, 873 articles were excluded based on the title and the abstract. This selection process resulted in 34 articles. After the full texts were read, 14 articles were excluded. The main reason the interventions were excluded was that they were not nursing interventions; if they were, they did not focus on the acute phase of hospital treatment or provide a perspective of the FMs. Finally, 20 original research articles were selected for review after completing the data retrieval process and the parallel analysis. Figure 1 illustrates the search selection process using a PRISMA flowchart.

### Description of Included Studies

Table 3 presents the selected research articles and highlights the studies’ characteristics and quality. Overall, the majority of the selected studies used a qualitative design (*n* = 10): Abrahamson et al., 2017; Adams & Dahdah, 2016; Degeneffe & Bursnall, 2015; Gan et al., 2010; Holloway et al., 2019; Keenan & Joseph, 2010; Kreitzer et al., 2019; Lefebvre & Levert, 2012a, 2012b; and Schutz et al., 2017. A cross-sectional design was used in eight research reports: (Arango-Lasprilla et al., 2010; Calvete & Arroyabe, 2012; Choustikova et al., 2020; de Goumoëns et al., 2019; Dillahunt-Aspillaga et al., 2013; Doyle et al., 2013; W. Liu et al., 2015; and Norup et al., 2015). The remaining studies used a mixed-methods design (*n* = 2): (Bellon et al., 2015; Kanmani & Raju, 2019).

**Table 3.** Characteristics of the Studies Selected in This Review (n = 20).

Author(s), year, country	Study design	Aim of study	Methods, participants and analysis	Main results	Quality score
Abrahamson et al. (2017), United Kingdom	Qualitative study	To explore the experiences of individuals who have had a severe traumatic brain injury (TBI) and their caregivers in the first month post-discharge from in-patient rehabilitation to living in the community.	Narratives of 10 patients and nine caregivers. Semi-structured interviews approximately 1 month post-discharge. Thematic analysis.	Patients and caregivers felt unsupported in the in-patient phase, during transitions between units, and when preparing for discharge; patients and caregivers struggled to accept a new reality of changed abilities, loss of roles, and loss of autonomy; early experiences post-discharge exacerbated fears for the future.	9/10^a^
Adams and Dahdah (2016), United States	Qualitative study	To explore adult TBI survivors’ and primary caregivers’ needs and deficits and to identify their self-initiated coping and adaptive strategies.	11 TBI patients and 6 primary caregivers (*N* = 17). Semi-structured interviews. Thematic analysis.	TBI survivors and caregivers identified patience and understanding, support, and professional help as their most relevant needs. Participants offered suggestions for mental health professionals to address how to work with brain injury survivors and their primary caregivers more effectively.	7/10^a^
Arango-Lasprilla et al. (2010), United States	A cross-sectional descriptive study	To determine the most and least important family needs in a group of family caregivers of individuals with TBI from Cali, Colombia, and to examine which of those needs were more likely to be met and unmet.	Twenty-nine family caregivers of individuals with TBI. The Family Needs Questionnaire was used in data collection. Statistical analysis.	Health Information, Community Support Network, and Professional Support Network sub-scales indicated the most important needs this group of Colombian TBI family caregivers reported. The most frequently met needs in the present study fell within Health Information, Involvement with Care, and Instrumental Support sub-scales, and the most frequently unmet needs fell within the Emotional Support, Instrumental Support, and Professional Support sub-scales.	6/8^b^
Bellon et al. (2015), Australia	Mixed-methods design, including postal survey and focus groups	To identify and compare family support needs following an acquired brain injury (ABI) in metropolitan and regional/remote areas to inform the development of a state-wide family peer support network.	The survey was completed by 194 family members (FMs) who provide support to an adult with an ABI. Focus groups included 43 participants (29 FMs, 14 ABI people). Thematic analysis of open-ended survey responses and focus group transcripts revealed 15 areas of needed support.	A strong focus was placed on the need for counseling and emotional support as well as family support groups for participants in major cities and regional/remote areas. Each support was reviewed to identify those that could be augmented through peer support, including emotional support, family support groups, ABI information, family social activities, help navigating the system, early supports (within the first year after the ABI), and self-advocacy training.	6/10^a^
Calvete and Arroyabe (2012), Spain	Cross-sectional study	To examine the associations between social support, coping responses, and depressive and grief symptoms in caregivers of people with a TBI.	The study included 223 caregivers (72.2% female and 26.9% male) from Spain. Family Needs Questionnaire, Texas Revised Inventory of Grief, Center for Epidemiological Studies Depression Scale, and the Responses to Stress Questionnaire. Statistical analysis.	A structural equation model indicated that secondary control coping was associated with less grief and depressive symptoms whereas primary control coping and disengagement were associated with more symptoms. Emotional and instrumental supports were directly associated with less depressive symptoms. In addition, emotional and professional support was associated with symptoms through primary control and disengagement coping.	5/8^b^
Choustikova et al. (2020), Finland	Cross-sectional study	To examine TBIs and patients’ FMs’ experiences of the support they received from health care professionals in acute care hospitals.	The study included 102 TBI patients’ FMs from Finland. A structured questionnaire was developed and used in the data collection. Statistical analysis.	A factor analysis revealed five factors that describe the guidance of TBI patients’ FMs: guidance of TBI patients’ symptoms and survival, benefits of guidance, needs-based guidance, guidance for the use of services, and guidance methods. Most of the FMs (51%–88%) felt that they had not received enough guidance from health care professionals in acute care hospitals across all five support aspects.	4/8^b^
de Goumoëns et al. (2019), Switzerland	Cross-sectional study	To identify and compare the needs of families of patients with an ABI in acute care and rehabilitation settings.	Data were collected in the acute care setting and in the rehabilitation setting during meetings with families (*n* = 54) of patients with ABI using the Family Needs Questionnaire. Statistical analysis.	In both settings, families provided information about the ABI or the patients’ health as the most important need, followed by support from health care professionals.	6/8^b^
Degeneffe and Bursnall (2015), United States	Qualitative study	To explore what adult siblings found beneficial and in need of improvement with the TBI professional services their injured brother or sister and family received.	The study included 267 TBI patients’ FMs (adult siblings’) views of professionals’ competence and received services. Constant comparative analysis.	Four interconnected themes: inadequate system-level response, lack of professional skills and understanding, lack of information provided, and beneficial and effective services. The siblings’ comments suggested that the system-level response to people with TBIs and their families was inadequate, that many professionals lacked the skills and understanding to provide effective services, and that professionals did not provide sufficient information to people with a TBI or their families.	6/10^a^
Dillahunt-Aspillaga et al. (2013), United States	Cross-sectional study	To acquire new knowledge about the impact that caring for individuals with a TBI has on FMs who are caregivers and to identify the critical resources and support these families in Florida need.	The study included 53 TBI patients’ caregivers. A structured questionnaire (BIAF Caregiver Needs Assessment Survey). Statistical analysis.	Caregivers of individuals with TBI in Florida highlighted critical resources and support, including long-term social, emotional, educational, informational, and financial needs. These findings illustrate the importance of following caregivers of individuals with a TBI after discharge from acute care. Findings also highlight that many caregivers may not report needs or concerns when providing care for people with a TBI.	5/8^b^
Doyle et al. (2013), United States	Cross-sectional study	Examined relationships between caregivers’ mental health and the extent to which needs were met in families of individuals with a TBI in Mexico City, Mexico.	The study included 68 TBI patients’ caregivers. Family Needs Questionnaire (FNQ) and Satisfaction with Life Scale (SWLS). Statistical analysis.	Twenty-seven percent of caregivers reported clinically significant depression levels, 40% reported below-average life satisfaction, and 49% reported mild-to-severe burden. Several of the most frequently met family needs were in the emotional support domain, whereas most unmet needs were in the health information domain. Family needs and caregiver mental health were significantly and highly related.	5/8^b^
Gan et al. (2010), Canada	Qualitative study	To achieve a rounded, multi-layered understanding of caregivers’ support needs.	39 caregivers across urban and rural settings in Ontario participated in focus groups. Interviews. Content analysis.	Five themes were formed: coping, supports that worked, supports needed, barriers, and ideal-world recommendations. This convergence of evidence underscores that caregiver support needs to transcend geographical boundaries and must be comprehensive, accessible, and long-term and encompass education, emotional, and instrumental support.	7/10^a^
Holloway et al. (2019), United Kingdom	Qualitative study	To explore how families are affected and integrate their views on the formal/informal support received as a consequence of an ABI.	This study included 16 FMs of people with a severe ABI. Semi-structured interviews. Inductive thematic analysis.	FMs’ experiences are complex, enduring, and affected by the context in which the ABI occurs as well as by formal/informal support. The grief FMs’ experience is ambiguous and develops over time, and they perceive few options but to remain involved. Experience of formal and informal support varies significantly in availability and quality, and poor support exacerbates difficulties and isolates FMs.	8/10^a^
Kanmani and Raju (2019), India	Mixed-methods design	To explore caregivers’ psychosocial distress and concerns in the emergency and trauma care (ETC) setting.	This study included 50 caregivers. Face‑to‑face interviews, focus group (over 6 months), and Depression Anxiety and Stress scale (DASS‑21) were used in data collection. Thematic analysis and statistical analysis.	In the quantitative analysis, caregivers’ mean age was found to be 45 (*M* = 45.00 ± 13.83) years. Caregivers had experienced mild depression (13.36 ± 3.07), moderate anxiety (13.70 ± 3.03), and minimal stress (13.66 ± 2.98) levels. Qualitative results revealed the following themes: difficulty in accessing timely care, uncertainty about the prognosis and future, family concerns and financial constraints, personal feelings, personal needs, and supportive care. A Chi‑square test revealed no significant association among caregivers’ gender and depression (χ^2^ = 2.381, *p* < .12), anxiety (χ^2^ = 0.01, *p* < .92), and stress (χ^2^ = 0.235, *p* < .61) levels.	6/10^a^
Keenan and Joseph (2010), Canada	Qualitative study	To identify the needs FMs expressed as patients with severe brain injury progress through their recovery.	This study included 25 FMs who were associated with 15 injured relatives. Data were collected from 44 interviews conducted at two time periods: discharge from ICU (Time 1) and discharge from acute care facility to home or rehabilitation (Time 2). Thematic analysis.	At Time 1, the researchers identified four main themes that described the trajectory of the families’ experiences: getting the news, uncertainty, making sense of the news, and moving on. At Time 2, themes of the families’ experience included uncertainty, looking for progress, transition, and letting go/building a new connection. Support the family required included the need for information, professional support, and community support. Families had intensive needs in the acute phase of the injury, and their needs changed over time.	7/10^a^
Kreitzer et al. (2019), United States	Qualitative study	To describe informal caregivers’ unmet needs.	Eighteen patient-caregiver dyads were enrolled. Fifty-three interviews with caregivers were completed. Semi-structured interviews with informal caregivers of moderate and severe TBI survivors were conducted 72 hr, 1 month, 3 months, and 6 months after injury. Thematic analysis.	Three themes were identified in the qualitative analysis: caregiver burden, caregiver health-related quality of life, and caregiver needs for information and support.	6/10^a^
Lefebvre and Levert (2012a), Canada	Qualitative study	To explore the needs of individuals with TBIs and their loved ones throughout the continuum of care and services.	The data were collected from focus groups with 150 participants (individuals with TBIs, their loved ones, and health care professionals) divided into 18 focus groups. Interviews. Thematic content analysis.	Despite regional differences, the results demonstrate participants’ very similar perceptions regarding their needs such as information, support, and a collaborative relationship with health care professionals individuals with TBIs and their loved ones experienced. These needs change throughout the stages of care. The fulfillment of these needs plays a determining role throughout the adaptation process of individuals with TBIs and their loved ones. Health care professionals must adopt a personalized approach to respond to needs related to the evolution of information, support, and relationships.	5/10^a^
Lefebvre and Levert (2012b), Canada	Qualitative study	To paint a picture of the needs of people close to individuals with a TBI and the services offered to answer these needs, from the point of view of the individuals with a TBI and health care professionals.	The sample comprised Montreal FMs (*n* = 4), Outaouais FMs (*n* = 8), Abitibi FMs (*n* = 7), Montreal care providers (*n* = 9), Outaouais care providers (*n* = 11), and Abitibi care providers (*n* = 9). DRAP (developing reflexive analysis for partnership) was used as a data collection method. Thematic content analysis.	The results show that people close to individuals with a TBI need information on the health problem, specifically regarding the diagnosis, the prognosis, and the factors that influence it as well as the steps toward rehabilitation, care, and services. The results show that close ones need specific, quality services and continuity of services.	7/10^a^
W. Liu et al. (2015), China	Cross-sectional study	To evaluate the impact of the varying severity of the TBI’s effect on family caregivers’ psychological state and demands.	Three hundred caregivers related to TBI victims were randomly selected. The Symptom Checklist-90 (SCL-90) was used to assess family caregivers’ psychological statuses, and the Critical Care Family Needs Inventory (CCFNI) was used to determine family caregivers’ needs. Statistical analysis.	SCL-90 scores for each psychological dimension were significantly higher with increased TBI severity (*p* < .05). Similarly, CCFNI scores were significantly higher with increased TBI severity (*p* <.05) for information, reassurance, and accessibility. These same dimensions were the most important needs for FMs of TBI injury victims, and support and comfort were the least important dimensions.	5/8^b^
Norup et al. (2015), Denmark	Cross-sectional study	The objective of this study was to explore differences by country in the importance of family needs after a TBI and differences in met/unmet needs.	Two hundred and seventy-one FMs of an individual with a TBI from Mexico, Colombia, Spain, Denmark, and Norway Family Needs Questionnaire. Statistical analysis.	Eight of the ten needs rated as most important globally were from the Health Information subscale. Importance ratings on the Health Information, Professional Support, and Involvement With Care subscales were similar across countries, but Mexican FMs rated Instrumental Support needs as less important than Colombian, Spanish, and Danish FMs. They also rated their Community Support needs as less important than Danish and Spanish FMs. Mexican FMs rated emotional support needs as less important than Colombian, Spanish, and Danish FMs. Globally, the needs rated as most often met were from the Health Information subscale, and the most unmet needs were from the Emotional Support subscale.	6/8^b^
Schutz et al. (2017), United States	Qualitative study	To explore how FMs, nurses, and physicians experience the palliative and supportive care needs of patients with severe acute brain injury (SABI) receiving care in the neuroscience intensive care unit (neuro-ICU).	Thirty-bed neuro-ICU in a comprehensive regional stroke and level-one trauma center in the United States. 47 completed interviews regarding 15 patients with FMs (*n* = 16), nurses (*n* = 15), and physicians (*n* = 16). Semi-structured interviews Thematic analysis.	Two themes were identified: (a) hope and (b) personhood. (a) Families linked prognostic uncertainty to a need for hope and expressed a desire for physicians to acknowledge this relationship. The language of hope varied depending on the participant: clinicians used hope as an object that can be given or taken away, generally in the process of conveying a prognosis, and families expressed hope as an action that helped them cope with their loved one’s acute illness and its prognostic uncertainty. (b) Participants described the loss of personhood through brain injury, the need to recognize and treat the brain-injured patient as a person, and the importance of relatedness and connection, including clinicians’ personal support of families.	7/10^a^

*Note.* TBI = traumatic brain injury; ABI = acquired brain injury; BIAF = The Brain Injury Association of Florida.

a Qualitative study = Quality score of JBI (Joanna Briggs Institute) critical appraisal checklist, including 10 criteria to assess the methodological quality of qualitative studies. ^b^Cross-sectional study = Quality score of JBI (Joanna Briggs Institute) critical appraisal checklist, including 8 criteria to assess the methodological quality of cross-sectional studies.

Most studies were conducted in the United States (*n* = 7), Canada (*n* = 4), and the United Kingdom (*n* = 2). The remaining seven studies were from Australia (*n* = 1), Spain (*n* = 1), Finland (*n* = 11), Switzerland (*n* = 11), India (*n* = 11), China (*n* = 1), and Denmark (*n* = 1). Most studies focused on FMs’ experiences of empowering support (*n* = 15). However, five studies discussed the perspective of empowerment more broadly, such as from the perspective of TBI patients and health care professionals.

### Synthesis of Results

**Figure 2.**
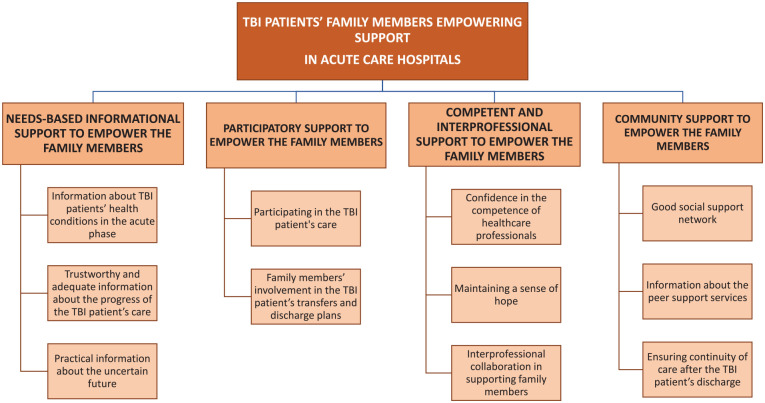
Illustration of Traumatic Brain Injury Patients’ Family Members’ Empowerment in Acute Care Hospitals.

Data synthesis with an integrated approach was used. Based on convergent results of the systematic literature review, empowering support for FMs in the acute phase of TBI patient hospital care is based on four main themes of the empowerment process: (a) needs-based informational support, (b) participatory support, (c) competent and interprofessional support, and (d) community support (see Figure 2).

#### Theme 1: Needs-Based Informational Support to Empower the FMs

The FMs’ most pressing need was identified as a need for information during the acute phase of TBI patients’ hospital care, which lasted throughout the patient’s hospital treatment, from emergency care to discharge (Keenan & Joseph, 2010; Kreitzer et al., 2019; Lefebvre & Levert, 2012a). However, FMs’ needs and ability to acquire information changed over time (Keenan & Joseph, 2010; Lefebvre & Levert, 2012a). For example, during emergency care and intensive care, the members of the family needed information focused on the TBI patient’s health conditions, medical treatment, and recovery (Doyle et al., 2013; Lefebvre & Levert, 2012a; W. Liu et al., 2015). In the inpatient ward, the FMs’ need for information focused more on practical issues and future plans (Keenan & Joseph, 2010; Lefebvre & Levert, 2012a). Although the FMs’ need for information changed over time, to empower the FMs, the information needed be trustworthy, versatile, and consistent (de Goumoëns et al., 2019; Gan et al., 2010; Lefebvre & Levert, 2012b).

Needs-based informational support to empower FMs contained three sub-themes: information about TBI patients’ health conditions in the acute phase, trustworthy and adequate information about the progress of the TBI patients’ care, and practical information about the uncertain future.

Information about TBI patients’ health conditions in the acute phase described the importance for FMs to have an early diagnosis of the patient’s brain injury (Choustikova et al., 2020; Gan et al., 2010). FMs wished to receive factual information about the accident (Keenan & Joseph, 2010), the brain injury, and its effect on the future (Bellon et al., 2015) at an early stage (Kanmani & Raju, 2019). If FMs felt they were receiving too little information from health care professionals, they would seek more information online or from their friends and relatives (Lefebvre & Levert, 2012a). Receiving sufficient information about the symptoms of TBI such as memory disorders (Arango-Lasprilla et al., 2010), emotional problems (Abrahamson et al., 2017), and changes in mood and personality (Adams & Dahdah, 2016; Calvete & Arroyabe, 2012; Gan et al., 2010; Kreitzer et al., 2019), helped FMs to understand, for example, why their relative with TBI displayed changes in behavior (Degeneffe & Bursnall, 2015). In addition, FMs wished to receive information on the TBI patient’s medical care (Doyle et al., 2013; Lefebvre & Levert, 2012a; W. Liu et al., 2015), and they needed reassurance that the patient received all necessary medical care (Gan et al., 2010).

FMs wanted trustworthy and adequate information about the progress of the TBI patient’s care, such as any changes in the TBI patient’s condition, and that all questions were answered honestly (Kreitzer et al., 2019; Lefebvre & Levert, 2012b) and professionally (Keenan & Joseph, 2010). FMs hoped that hospital staff would always be honest with them, even when the patient’s condition worsened. The research demonstrated that honesty was seen as a characteristic of professionalism that promoted the development of a trusting relationship between FMs and health care professionals (Keenan & Joseph, 2010; Kreitzer et al., 2019; Lefebvre & Levert, 2012b). FMs could better understand the purpose of their relative’s care if the information was conveyed in a peaceful environment with sufficient processing time (de Goumoëns et al., 2019). The information also needed to be provided in oral form (Gan et al., 2010; Lefebvre & Levert, 2012b) and written form (Choustikova et al., 2020). In addition, from the empowerment perspective, FMs wished to receive regular patient updates (Keenan & Joseph, 2010; Lefebvre & Levert, 2012b) that were specific to their relative and not based on general statistics and probabilities in order to utilize the information in their decision-making (Keenan & Joseph, 2010).

FMs needed practical information about the uncertain future after the TBI patient had left the ICU and the situation had stabilized (Lefebvre & Levert, 2012a). FMs’ needs for information shifted from damages the accident caused and medical care to planning for the future (Keenan & Joseph, 2010). In the inpatient ward, FMs’ needs focused on receiving sufficient guidance (Choustikova et al., 2020) and support for practical issues such as organizing extended hospital visits and managing financial matters (Abrahamson et al., 2017). At this point, FMs started to realize they had to attend to other obligations such as family (Adams & Dahdah, 2016; Arango-Lasprilla et al., 2010), work, and community life (Keenan & Joseph, 2010). FMs frequently wondered how TBI would affect the patient’s life in the areas of work (Calvete & Arroyabe, 2012), independence (Degeneffe & Bursnall, 2015), family activities (Dillahunt-Aspillaga et al., 2013; Gan et al., 2010; Holloway et al., 2019) and marriage (Lefebvre & Levert, 2012a). FMs also needed support and information about taking care of themselves, for example, by taking a break from the care, problems, and responsibilities (Doyle et al., 2013). In the inpatient ward, FMs were interested in finding out about available services (Kreitzer et al., 2019) and resources (Adams & Dahdah, 2016) to ease their social adaptation as well as to promote the family’s independence and coping after hospital discharge (Lefebvre & Levert, 2012a).

#### Theme 2: Participatory Support to Empower the FMs

Uncertainty and concern about the patient’s survival increased the FMs’ feelings of powerlessness and, arguably, their need to participate in the patient’s care (Bellon et al., 2015; de Goumoëns et al., 2019; Keenan & Joseph, 2010). To empower the FMs, the professionals must recognize them as an integral part of the TBI patient’s comprehensive nursing process (Degeneffe & Bursnall, 2015). Being close to the patient was the primary way for FMs to participate in the patient’s care (Keenan & Joseph, 2010). However, concretely participating in the patient’s care through the nursing procedures and the patient’s transfers and discharge plans was also important for empowering FMs (Lefebvre & Levert, 2012a; W. Liu et al., 2015; Norup et al., 2015).

This theme included two sub-themes: participating in the TBI patient’s care and FMs’ involvement in the TBI patient’s transfers and discharge plans.

By participating in the TBI patient’s care, FMs reported feeling part of the patient’s holistic care (Calvete & Arroyabe, 2012) and nursing process (Degeneffe & Bursnall, 2015). This, in turn, promoted the FMs’ understanding of the situation and future (Bellon et al., 2015; de Goumoëns et al., 2019) and helped to identify their abilities, to trust in themselves, and their coping process at home (Calvete & Arroyabe, 2012; Lefebvre & Levert, 2012a). In addition to practical duties (e.g., assisting in washing and eating), participating in planning and decision-making were considered essential aspects of inclusion in the patient’s care (Bellon et al., 2015; de Goumoëns et al., 2019). However, just staying at the patient’s side was enough to create a sense of participation (Calvete & Arroyabe, 2012; Kanmani & Raju, 2019). Being at the patient’s side increased FMs’ sense of managing the situation and created an optimistic feeling that their relative’s recovery was progressing (Keenan & Joseph, 2010).

FMs’ involvement in the TBI patient’s transfers and discharge plans was significant for FMs. They wanted to participate in planning the discharge together with the professionals (Lefebvre & Levert, 2012a; W. Liu et al., 2015; Norup et al., 2015) because FMs usually knew better if the patient could cope at home and whether the necessary preparations had been made at home (Abrahamson et al., 2017). Problems with hospital discharges are often related to poor communication, inadequate planning, and abrupt discharges without prior notice to the FMs (Abrahamson et al., 2017). Delays and long waiting times for transport without timely provision of information exacerbated anxiety (Abrahamson et al., 2017) and perceived burden (Kreitzer et al., 2019) among FMs. Proactive discharge planning, identifying differences between units (Keenan & Joseph, 2010), evaluating the FMs’ and patient’s needs, and setting goals together with nursing staff (Abrahamson et al., 2017) reduced the anxiety experienced by FMs (Keenan & Joseph, 2010). It enhanced their preparedness to cope at home (Calvete & Arroyabe, 2012).

#### Theme 3: Competent and Interprofessional Support to Empower the FMs

The versatile support from health care professionals was one of the essential factors in empowering FMs during the acute phases of the TBI patient’s treatment. To empower the FMs, the health care professionals needed to be competent, listen, and maintain the FMs’ sense of hope throughout the patient’s treatment (Abrahamson et al., 2017; de Goumoëns et al., 2019; Gan et al., 2010; Kreitzer et al., 2019; W. Liu et al., 2015). The nurse’s role was especially significant in empowering FMs because they were often considered to be part of the family (Keenan & Joseph, 2010). In addition, participating in interprofessional collaboration to support FMs was also perceived as a significant factor in empowering families because their needs changed during the different phases of the patient’s hospital care (Choustikova et al., 2020; Keenan & Joseph, 2010; Lefebvre & Levert, 2012b).

This theme included three sub-themes: confidence in the competence of health care professionals, maintenance of a sense of hope, and interprofessional collaboration to support FMs.

The FMs’ confidence in the competence of health care professionals was necessary (Gan et al., 2010) because it increased their feeling that the patient was receiving holistic care (W. Liu et al., 2015). Professionals’ knowledge and skills in caring for the TBI patient demonstrated the staff’s competence. This and communication were the key factors influencing the FMs’ experience receiving empowering professional support (Keenan & Joseph, 2010). It was important to ensure that FMs were able to talk to a doctor at least once a day; otherwise, the FMs experienced disappointment (Calvete & Arroyabe, 2012). In this study, the health care professionals who expressed little interest in involving the family were perceived as leaving the FMs alone with difficult issues. Talking about difficult issues with professionals eased the FMs’ fear, anxiety, and shock (Choustikova et al., 2020). In addition, having a good relationship with professionals allowed the FMs to feel that they were part of the team, the treatment, and the decision-making process (Lefebvre & Levert, 2012b).

Furthermore, good communication and information sharing between FMs, and staff promoted the coordination of care and achievement of shared goals (de Goumoëns et al., 2019). The need for cohesive, consistent, and long-term communication between service providers and between service providers and families was essential for empowering FMs (Abrahamson et al., 2017; de Goumoëns et al., 2019; Kreitzer et al., 2019).

To empower the FMs, health care professionals require good listening skills (Calvete & Arroyabe, 2012), know the family, and communicate with different health care providers (Keenan & Joseph, 2010). FMs wished to be heard more on patient-related issues (Kreitzer et al., 2019) because they felt they had valuable (Lefebvre & Levert, 2012b) and useful (Holloway et al., 2019) knowledge to convey that could prevent the staff from making false conclusions (Choustikova et al., 2020). Especially in situations where the patient had limited communication ability, involving the family was an important factor for the patient’s recovery (Holloway et al., 2019) and the FMs’ adaptation (de Goumoëns et al., 2019).

Maintaining a sense of hope was needed because unexpected news of an accident causes a powerful emotional reaction (Bellon et al., 2015; Keenan & Joseph, 2010) and a sense of powerlessness among FMs (Lefebvre & Levert, 2012a). Uncertain prognosis of the TBI increased the FMs’ need for hope (W. Liu et al., 2015), and they wished for health care professionals to recognize this association (Schutz et al., 2017). Although the FMs wanted truthful information, they also wanted health care professionals to give them hope for the future (W. Liu et al., 2015). Even in cases of patient death, the FMs remained hopeful and focused on minimizing the perceived suffering of the TBI patient (Schutz et al., 2017). This sense of hope gave FMs the strength to ensure their loved ones received the best care possible (Calvete & Arroyabe, 2012). However, FMs need professional encouragement (W. Liu et al., 2015) to maintain a sense of hope (Arango-Lasprilla et al., 2010). Physicians were perceived as being particularly pessimistic (Keenan & Joseph, 2010), emphasizing nurses’ role in maintaining hope and empowerment for the FMs (Schutz et al., 2017).

In the acute phase of hospital care, FMs had many questions (Norup et al., 2015) and challenges (Abrahamson et al., 2017); thus, interprofessional collaboration in supporting FMs was needed (Keenan & Joseph, 2010; Lefebvre & Levert, 2012b). For example, FMs wanted to see a hospital chaplain to discuss and share their feelings (W. Liu et al., 2015) and to meet a social worker to handle financial matters (Choustikova et al., 2020). Many FMs also hoped to meet with a physiotherapist and psychiatric nurse during the acute phase of hospital care (Choustikova et al., 2020). In addition, FMs needed interprofessional support in planning the future to strengthen their sense of control over the new situation at home with the TBI survivors, which usually arose from insecurities FMs experienced due to the potentially progressive nature of TBIs (Gan et al., 2010).

#### Theme 4: Community Support to Empower the FMs

The findings highlight community support as a fundamental part of empowering FMs. Arguably, it is essential for FMs to receive support from health care professionals, FMs, and friends (Calvete & Arroyabe, 2012; Holloway et al., 2019; Keenan & Joseph, 2010). In addition, the results indicate that peer support services complement the support for FMs and reduce the FM’s feelings of anxiety and fear (Gan et al., 2010; Norup et al., 2015). However, at the end of the patient’s treatment, the FMs hoped the patient’s treatment would continue after hospitalization. Once again, the nurses’ role was emphasized because the FMs hoped that the nurses would coordinate the follow-up care and organize the services. In summary, community support can empower FMs in the long term (Abrahamson et al., 2017; Bellon et al., 2015; Doyle et al., 2013; Lefebvre & Levert, 2012a; W. Liu et al., 2015; Norup et al., 2015).

This theme had three sub-themes: good social support network, information about peer support services, and ensuring continuity of care after the TBI patient’s hospital discharge.

A good social support network meant tangible help was available from friends or relatives, such as when transporting the family to the hospital (Calvete & Arroyabe, 2012) or taking care of the children’s needs (Keenan & Joseph, 2010). However, the mere presence of friends and other FMs (Calvete & Arroyabe, 2012) made the FMs feel they were not alone with all the challenges and thus promoted their feeling of empowerment (Holloway et al., 2019; Keenan & Joseph, 2010). Despite welcoming community support, FMs also wanted health care professionals to address the burden that the number of contacts from relatives caused (Calvete & Arroyabe, 2012). FMs perceived time spent with friends and relatives and answering their questions as cumbersome and stressful. They wanted to have professional guidance (Lefebvre & Levert, 2012a) and support (Keenan & Joseph, 2010) to limit their contacts (Calvete & Arroyabe, 2012).

FMs were interested in obtaining information about different peer support services at the first stage of hospitalization (Norup et al., 2015). Peer support services offer timely and helpful support in a crisis and relevant information on various resources for FMs (Gan et al., 2010). To be empowered, FMs needed to share their feelings (Norup et al., 2015) and experiences (Bellon et al., 2015) with people who had been in the same situation and had faced the same problems. Thus, they could offer suggestions and solutions for arising issues (Gan et al., 2010) and help them prepare for the worst (Arango-Lasprilla et al., 2010). Other people’s stories and experiences about the effects of TBI on family life gave FMs courage (Gan et al., 2010). They made them feel hopeful about the TBI patient’s recovery (Keenan & Joseph, 2010) and the family’s coping (Adams & Dahdah, 2016).

Ensuring continuity of care after the TBI patient’s hospital discharge was critical. At the end stage of the patient’s inpatient care, FMs hoped there was a person who would manage and coordinate the discharge and organization of services (Bellon et al., 2015; Lefebvre & Levert, 2012a; W. Liu et al., 2015; Norup et al., 2015). FMs hoped for a nurse to assume the responsibility for coordinating duties, providing information, and organizing the necessary care meetings and services (Abrahamson et al., 2017; W. Liu et al., 2015) because FMs frequently experienced inequalities in access to services (Bellon et al., 2015; Holloway et al., 2019). Access to necessary support services was crucial for empowering FMs because studies have shown such services promote FMs’ adaptation to their new roles, ease intrafamilial relationships, satisfy families’ long-term needs (Bellon et al., 2015; Gan et al., 2010), and reduce the sense of burden FMs experienced (Doyle et al., 2013).

## Discussion

### Summary of Findings

The findings of this systematic review outline the factors contributing to the empowering support of FMs while also describing the empowerment support from the FMs’ perspective during the acute phase of hospital care. We have defined the process of empowerment as a dialogical and supportive relationship between FMs and health care professionals, in which the FMs were seen as part of the TBI patients’ comprehensive treatment planning and implementation throughout the acute hospitalization period. Needs-based informational, participatory, professional, and community support were identified as factors of the empowerment process to promote FMs’ empowerment.

FMs are empowered when they have sufficient, concrete, and needs-based information about brain injury, its treatment, and its effect on the future from health care professionals during the acute phase of care. This enabled the FMs to utilize information in their decision-making and hence better process the consequences and effects of brain injury on family activities (de Goumoëns et al., 2019; W. Liu et al., 2015). FMs’ needs for information change in time (Lefebvre & Levert, 2012a) and, according to Keenan and Joseph (2010), decrease by 50% when the patient is transferred from the ICU to an inpatient ward. Later in the inpatient ward, FMs felt more capable of evaluating the progress of the patient’s recovery (Keenan & Joseph, 2010). At this point, it was important for the FMs to become informed about practical factors, such as transport services and managing finances (Abrahamson et al., 2017). From the empowerment perspective, the results support the findings of Wåhlin’s (2017) research, which identified knowledge as an empowerment-promoting tool and receiving information as an integral part of it. However, another point to consider is that the quality of the information and the environment where the information is offered also affects the extent of FMs’ empowerment. The information should thus be tailored to fit the FMs’ needs. The closer the received information and support are to the FMs’ needs, the more potential there is for empowerment (Funnell et al., 1991). In light of this new knowledge, future health care professional education should focus on how to offer guidance, especially from the perspective of the family’s needs (Choustikova et al., 2020). The International Family Nursing Association (IFNA, 2017) has developed advanced practice competencies for family nursing that may be useful for health care professionals working with this population of families.

Participating in the patient’s care and involvement in the patient’s transfers and discharge plans were also associated with empowering FMs (Lefebvre & Levert, 2012a; Norup et al., 2015), as it made the FMs feel they were useful and part of the patient’s holistic care (Calvete & Arroyabe, 2012). This further corroborates previous results (Kivunja et al., 2018; Manskow et al., 2017), although Oyesanya (2017) found that FMs frequently felt they were invading the health care staff’s territory by actively participating in the patient’s care. However, Wetzig and Mitchell (2017) discovered that health care professionals recognized the benefits and significance of FMs’ involvement from the perspective of the TBI patient’s recovery in acute care. The previous results emphasize that participation in patient care has also been essential in empowering the FMs because they are viewed as equal and active partners in TBI patient treatment (Degeneffe et al., 2011; Man et al., 2003; Rodwell, 1996). Thus, health care professionals should actively encourage and guide FMs on how to participate in patient care concretely. In addition, health care professionals should boldly involve FMs in all phases of the patient’s treatment plan and decision-making process to ensure that both the FMs and professionals have up-to-date information on future activities and plans and to allow the FMs to feel that they are a part of the patient’s nursing process (Lefebvre & Levert, 2012b).

Our results also show that FMs need competent professional support as well as interprofessional collaboration to comprehend the trauma (de Goumoëns et al., 2019). Thus, FMs can mourn the damage the brain injury caused; process (Keenan & Joseph, 2010), and manage (Abrahamson et al., 2017) their own emotions, such as fear, grief, anger, and guilt (Calvete & Arroyabe, 2012); and adjust to the new life situation (Lefebvre & Levert, 2012a). Health care professionals, especially nurses (de Goumoëns et al., 2019), played a significant role in supporting the FMs of TBI patients during the acute phase of hospital care (Keenan & Joseph, 2010). Earlier studies have confirmed an equal and trustful communication between health care professionals and FMs can contribute to empowering the latter (Sigurdardottir et al., 2015) and decrease the feelings of burden (Sakanashi & Fujita, 2017) and abandonment (Wåhlin, 2017). The empowered FMs feel emotionally and physically balanced, which increases confidence to act as a caregiver and further supports adaptation to a new situation (Sakanashi & Fujita, 2017). Even though empowerment cannot be handed over (Sigurdardottir et al., 2015), the review shows that nurses should recognize the effect of their support and actions on family members’ long-term capacities and well-being, particularly their coping at home after the TBI patient’s discharge.

In addition, the findings highlight how valuable it is for FMs to obtain support from outside the hospital in the acute phase of TBI patient care, particularly from other FMs, friends, and peers. Furthermore, emotional and financial support from the FMs; work environment was also significant (Gan et al., 2010). Before discharging the patients from the hospital, FMs hoped to establish a single contact point between the family and the health care and social services (Holloway et al., 2019) to provide long-term support based on the FMs’ needs, which change with time (Abrahamson et al., 2017). Undoubtedly, the nurse’s role was significant again because the FMs hoped that the nurses would take responsibility for organizing follow-up and aftercare services (W. Liu et al., 2015). It was essential to ensure continuity of care and access to support services during the acute phase of patient treatment to maintain the FMs’ well-being and ability to cope (Abrahamson et al., 2017) because FMs frequently reported facing a fragmented (Gan et al., 2010) and inconsistent health care system (Kreitzer et al., 2019) after hospital care.

Earlier studies have consistently demonstrated that FMs of TBI patients often experience anxiety, depression, social isolation, and economic disruption after hospital care (Anke et al., 2020; Manskow et al., 2017; McIntyre et al., 2020). Specifically, deficiencies in organizing and ensuring the provision of aftercare services for the family in the discharge phase may cause this. Regarding these findings, receiving incomplete or little information about support services during the patient’s hospitalization also delayed access to them and reduced FMs’ adaptation to their new role and living situation Bellon and colleagues (2015). Therefore, health care professionals must ensure that support services are available for TBI patients before being discharged from the hospital (Abrahamson et al., 2017). Although studies have demonstrated the importance of ensuring and securing the continuity of care for FMs (Bellon et al., 2015; W. Liu et al., 2015), they also show that it has not been recognized as part of the empowerment concept. However, the review revealed that information about support services alone was insufficient to ensure continuity of care for TBI patients and empower FMs.

In summary, FMs experience long and difficult times during a TBI patients’ hospitalization, especially in the ICU. Moreover, FMs have needs during the acute phases of the patients’ care that may have long-reaching consequences, such as feelings of burden, reduced life satisfaction, and depression, if they are not met. The goal of empowering FMs is to promote and maximize the FMs’ ability to manage independently with the TBI patient after hospitalization and to increase FMs’ coping and well-being. Receiving high-quality, sufficient information, participating in the patient’s care and decision-making, holistic support from health care professionals, and ensuring the TBI patient’s care constitute essential elements in the FMs’ empowerment process. Considering these elements when facing FMs during the acute phase of a TBI patient’s care may ease the family’s transition from hospital to home and facilitate adjusting to the new life situation.

However, it should be noted that the FMs’ experiences and perceived needs during the acute phases of care are insufficient sources of information to offer empowering support. Therefore, it is important to define and examine the concept of empowerment in more depth from the perspective of acute care. It would be interesting to determine whether the FMs’ primary information and support needs resulted from the sudden and uncertain nature of the brain injury, the hectic environment of acute care, and the limited resources available to the health care professionals, or a combination of these factors.

### Strengths and Limitations

Our systematic literature review was performed systematically and comprehensively, and the data analysis was conducted using original data. The university library information specialist was consulted in the data retrieval process to improve the data’s coverage and reliability. In addition, two researchers performed a literature quality assessment in parallel and independently. This systematic literature review contributes beneficial knowledge on empowering support for FMs of TBI patients during the acute phase of hospital care from the empowerment perspective.

However, this study may have limitations due to the lack of available literature on FMs’ empowerment. Moreover, empowerment is a multidimensional concept, and in this study, it was generally observed on an individual level. However, information about the organizational and community levels would have also provided a more comprehensive understanding of the empowerment process. According to Wåhlin (2017), it is possible that health care professionals need to feel empowered in their professional role in order to empower FMs which was not addressed in this review. Nevertheless, it is notable to understand that it is the health care professionals who form the healthcare organization.

We did not find any clinical trials in nursing that focused on the effectiveness or efficiency of empowering TBI patients’ FMs. Therefore, other aspects of empowerment may not have been identified and may warrant more thorough research in the future. For example, tested interventions can be used to ensure and strengthen the empowerment of FMs, even after hospitalization. Although these findings are based on the perspective of FMs, the results of this study can assist health care professionals in identifying factors that help FMs process and utilize the provided support and information to control new, possibly insecure, situations. Future studies should focus more on the perspectives of health care staff when empowering FMs in acute care to gain a deeper and more holistic understanding of empowerment in the context of TBI patient care.

## Conclusion

This study provides a systematic overview of the factors contributing to FMs’ empowerment and describes the empowerment from the FMs perspectives. We can conclude that empowerment in the acute phase of TBI patient treatment consists of an interactive relationship between FMs and professionals, which includes professionals providing comprehensive information and support and ensuring that the patient’s care will continue after hospitalization. Consequently, the process of empowering FMs does not end when the TBI patient’s acute phase ends, but instead continues after hospitalization.

Nevertheless, it is clear that in the future, it is essential to study the concept of empowerment more at the organizational and community levels in the context of acute care and from the perspective of health care professionals. Although this review provides information on the nature of empowering support for FMs of TBI patients during the acute phases of care, this information is derived mainly from qualitative and cross-sectional studies. In the future, clinical trials in TBI nursing aiming to find concrete and effective means to increase and to support TBI patients’ and family’s empowerment are needed. It might prove beneficial for future studies to redirect the methodology and study design toward interventional studies to obtain more comprehensive information on aspects of FMs’ empowerment support in acute care.

## Supplemental Material

sj-pdf-1-jfn-10.1177_10748407231171933 – Supplemental material for Empowering Support for Family Members of Brain Injury Patients in the Acute Phase of Hospital Care: A Mixed-Methods Systematic Review Click here for additional data file. Supplemental material, sj-pdf-1-jfn-10.1177_10748407231171933 for Empowering Support for Family Members of Brain Injury Patients in the Acute Phase of Hospital Care: A Mixed-Methods Systematic Review by Julia Lindlöf, Hannele Turunen, Tarja Välimäki, Justiina Huhtakangas, Sofie Verhaeghe and Kirsi Coco in Journal of Family Nursing

sj-pdf-2-jfn-10.1177_10748407231171933 – Supplemental material for Empowering Support for Family Members of Brain Injury Patients in the Acute Phase of Hospital Care: A Mixed-Methods Systematic Review Click here for additional data file. Supplemental material, sj-pdf-2-jfn-10.1177_10748407231171933 for Empowering Support for Family Members of Brain Injury Patients in the Acute Phase of Hospital Care: A Mixed-Methods Systematic Review by Julia Lindlöf, Hannele Turunen, Tarja Välimäki, Justiina Huhtakangas, Sofie Verhaeghe and Kirsi Coco in Journal of Family Nursing

## References

[bibr1-10748407231171933] Abrahamson V. Jensen J. Springett K. Sakel M . (2017). Experiences of patients with traumatic brain injury and their carers during transition from inpatient rehabilitation to the community: A qualitative study. Disability & Rehabilitation, 39(17), 1683–1694. 10.1080/09638288.2016.1211755 27557977

[bibr2-10748407231171933] Adams D. Dahdah M . (2016). Coping and adaptive strategies of traumatic brain injury survivors and primary caregivers. NeuroRehabilitation, 39(2), 223–237. 10.3233/NRE-161353 27372358

[bibr3-10748407231171933] Ania-Gonzalez N. Olano-Lizarraga M. Vazquez-Calatayud M . (2022). Interventions to empower cardiorenal patients: A systematic review. Journal of Advanced Nursing, 78(2), 363–376. 10.1111/jan.15007 34363636

[bibr4-10748407231171933] Anke A. Røe C. Sigurdardottir S. Norup A. Soberg H. L. Arango-Lasprilla J. C. Manskow U. S . (2020). Family needs at one and two years after severe traumatic brain injury: A prospective study of changes and predictors. Brain Injury, 34(1), 89–97. 10.1080/02699052.2019.1682191 31647690

[bibr5-10748407231171933] Arango-Lasprilla J. C. Quijano M. C. Aponte M. Cuervo M. T. Nicholls E. Rogers H. L. Kreutzer J . (2010). Family needs in caregivers of individuals with traumatic brain injury from Colombia, South America. Brain Injury, 24(7), 1017–1026. 10.3109/02699052.2010.490516 20545455

[bibr6-10748407231171933] Aslam S. Emmanuel P . (2010). Formulating a researchable question: A critical step for facilitating good clinical research. Indian Journal of Sexually Transmitted Diseases and AIDS, 31(1), 47–50. 10.4103/0253-7184.69003 21808439 PMC3140151

[bibr7-10748407231171933] Bellon M. Crocker R. Farnden J. Gardner J. Sando S. Peterson C . (2015). Family support needs following acquired brain injury across metropolitan and regional/remote south Australia. Brain Impairment, 16(2), 131–144. 10.1017/BrImp.2015.17

[bibr8-10748407231171933] Calvete E. de Arroyabe E. L . (2012). Depression and grief in Spanish family caregivers of people with traumatic brain injury: The roles of social support and coping. Brain Injury, 26(6), 834–843. 10.3109/02699052.2012.655363 22583174

[bibr9-10748407231171933] Capizzi A. Woo J. Verduzco-Gutierrez M . (2020). Traumatic brain injury: An overview of epidemiology, pathophysiology, and medical management. The Medical Clinics of North America, 104(2), 213–238. 10.1016/j.mcna.2019.11.001 32035565

[bibr10-10748407231171933] Chen Y. C. Li I. C . (2009). Effectiveness of interventions using empowerment concept for patients with chronic disease: A systematic review. JBI Evidence Synthesis, 7(27), 1179–1233. 10.11124/jbisrir-2009-208 27819885

[bibr11-10748407231171933] Choustikova J. Turunen H. Tuominen-Salo H. Coco K . (2020). Traumatic brain injury patients’ family members’ evaluations of the social support provided by healthcare professionals in acute care hospitals. Journal of Clinical Nursing, 29(17–18), 3325–3335. 10.1111/jocn.15359 32497326

[bibr12-10748407231171933] Coco K. Tossavainen K. Jääskeläinen J. E. Turunen H . (2011). Support for traumatic brain injury patients’ family members in neurosurgical nursing: A systematic review. Journal of Neuroscience Nursing, 43(6), 337–348. 10.1097/jnn.0b013e318234ea0b 22089411

[bibr13-10748407231171933] Degeneffe C. E. Bursnall S . (2015). Quality of professional services following traumatic brain injury: Adult sibling perspectives. Social Work, 60(1), 19–27. 10.1093/sw/swu047 25643572

[bibr14-10748407231171933] Degeneffe C. E. Chan F. Dunlap L. Man D. Sung C . (2011). Development and validation of the Caregiver Empowerment Scale: A resource for working with family caregivers of persons with traumatic brain injury. Rehabilitation Psychology, 56(3), 243–250. https://doi:10.1037/a0024465 21787096 10.1037/a0024465

[bibr15-10748407231171933] de Goumoëns V. Didier A. Mabire C. Shaha M. Diserens K . (2019). Families’ needs of patients with acquired brain injury: Acute phase and rehabilitation. Rehabilitation Nursing, 44(6), 319–327. 10.1097/rnj.0000000000000122 29300227

[bibr16-10748407231171933] de Goumoëns V. Rio L. M. Jaques C. Ramelet A. S . (2018). Family-oriented interventions for adults with acquired brain injury and their families: A scoping review. JBI Evidence Synthesis, 16(12), 2330–2367. 10.11124/JBISRIR-2017-003410 30531483

[bibr17-10748407231171933] Dillahunt-Aspillaga C. Jorgensen-Smith T. Ehlke S. Sosinski M. Monroe D. Thor J . (2013). Traumatic brain injury: Unmet support needs of caregivers and families in Florida. PLOS ONE, 8(12), Article e82896. 10.1371/journal.pone.0082896 PMC3866264 24358236

[bibr18-10748407231171933] Doser K. Norup A . (2016). Caregiver burden in Danish family members of patients with severe brain injury: The chronic phase. Brain Injury, 30(3), 334–342. 10.3109/02699052.2015.1114143 26829640

[bibr19-10748407231171933] Doyle S. T. Perrin P. B. Díaz Sosa D. M. Espinosa Jove I. G. Lee G. K. Arango-Lasprilla J. C . (2013). Connecting family needs and TBI caregiver mental health in Mexico City, Mexico. Brain Injury, 27(12), 1441–1449. 10.3109/02699052.2013.826505 23957747

[bibr20-10748407231171933] Elo S. Kääriäinen M. Kanste O. Pölkki T. Utriainen K. Kyngäs H . (2014). Qualitative content analysis: A focus on trustworthiness. SAGE Open, 4(1), 1–10. 10.1177/2158244014522633

[bibr21-10748407231171933] Friend M. L. Sieloff C. L . (2018). Empowerment in nursing literature: An update and look to the future. Nursing Science Quarterly, 31(4), 355–361. 10.1177/0894318418792887 30223743

[bibr22-10748407231171933] Funnell M. M. Anderson R. M. Arnold M. S. Barr P. A. Donnelly M. Johnson P. D. Taylor-Moon D. White N. H . (1991). Empowerment: An idea whose time has come in diabetes education. Diabetes Educator, 17(1), 37–41. 10.1177/014572179101700108 1986902

[bibr23-10748407231171933] Gan C. Gargaro J. Brandys C. Gerber G. Boschen K . (2010). Family caregivers’ support needs after brain injury: A synthesis of perspectives from caregivers, programs, and researchers. NeuroRehabilitation, 27(1), 5–18. 10.3233/NRE-2010-0577 20634597

[bibr24-10748407231171933] Garcia-Sierra R. Fernandez-Castro J . (2018). Relationships between leadership, structural empowerment, and engagement in nurses. Journal of Advanced Nursing, 74(12), 2809–2819. 10.1111/jan.13805 30019477

[bibr25-10748407231171933] Grant M. J. Booth A . (2009). A typology of reviews: An analysis of 14 review types and associated methodologies. Health Information & Libraries Journal, 26(2), 91–108. 10.1111/j.1471-1842.2009.00848.x 19490148

[bibr26-10748407231171933] Holloway M. Orr D. Clark-Wilson J . (2019). Experiences of challenges and support among family members of people with acquired brain injury: A qualitative study in the UK. Brain Injury, 33(4), 401–411. 10.1080/02699052.2019.1566967 30663417

[bibr27-10748407231171933] International Family Nursing Association. (2017). IFNA Position Statement on Advanced Practice Competencies for Family Nursing. https://internationalfamilynursing.org/2017/05/19/advanced-practice-competencies/

[bibr28-10748407231171933] Jochems D. van Rein E. Niemeijer M. van Heijl M. van Es M. A. Nijboer T. Leenen L. P. H. Houwert R. M. van Wessem K. J . (2021). Incidence, causes and consequences of moderate and severe traumatic brain injury as determined by Abbreviated Injury Score in the Netherlands. Scientific Reports, 11(1), Article 19985. 10.1038/s41598-021-99484-6 PMC8497630 34620973

[bibr29-10748407231171933] Jones P. S. Winslow B. W. Lee J. W. Burns M. Zhang X. E . (2011). Development of a Caregiver Empowerment Model to promote positive outcomes. Journal of Family Nursing, 17(1), 11–28. 10.1177/1074840710394854 21343620

[bibr30-10748407231171933] Kanmani T. R. Raju B . (2019). Caregiver’s psychosocial concerns and psychological distress in emergency and trauma care setting. Journal of Neurosciences in Rural Practice, 10(1), 54–59. 10.4103/jnrp.jnrp_129_18 30765971 PMC6337970

[bibr31-10748407231171933] Keenan A. Joseph L . (2010). The needs of family members of severe traumatic brain injured patients during critical and acute care: A qualitative study. Canadian Journal of Neuroscience Nursing, 32(3), 25–35. 20865832

[bibr32-10748407231171933] Kellermeyer L. Harnke B. Knight S . (2018). Covidence and Rayyan. Journal of the Medical Library Association, 106(4), 580. 10.5195/jmla.2018.513

[bibr33-10748407231171933] Kivunja S. River J. Gullick J . (2018). Experiences of giving and receiving care in traumatic brain injury: An integrative review. Journal of Clinical Nursing, 27(7-8), 1304–1328. 10.1111/jocn.14283 29396883

[bibr34-10748407231171933] Kreitzer N. Bakas T. Kurowski B. Lindsell C. J. Ferioli S. Foreman B. Ngwenya L. Thomas S. Keegan S. Adeoye O . (2019). The experience of caregivers following a moderate to severe traumatic brain injury requiring ICU admission. Journal of Head Trauma Rehabilitation, 35(3), E299–E309. 10.1097/HTR.0000000000000525 PMC10346118 31479080

[bibr35-10748407231171933] Kreutzer J. S. Marwitz J. H. Klyce D. W. Schaaf K. P. W. Sima A. P. Welch A. M. Niemeier J. P . (2018). Family needs on an inpatient brain injury rehabilitation unit: A quantitative assessment. The Journal of Head Trauma Rehabilitation, 33(4), 228–236. 10.1097/HTR.0000000000000390 29601345

[bibr36-10748407231171933] Lefebvre H. Levert M. J . (2012a). The needs experienced by individuals and their loved ones following a traumatic brain injury. Journal of Trauma Nursing, 19(4), 197–207. 10.1097/JTN.0b013e318275990d 23222398

[bibr37-10748407231171933] Lefebvre H. Levert M. J . (2012b). The close relatives of people who have had a traumatic brain injury and their special needs. Brain Injury, 26(9), 1084–1097. 10.3109/02699052.2012.666364 22624724

[bibr38-10748407231171933] Liu G. Ou S. Cui H. Li X. Yin Z. Gu D. Wang Z . (2021). Head injury and amyotrophic lateral sclerosis: A meta-analysis. Neuroepidemiology, 55(1), 11–19. 10.1159/000510987 33621971

[bibr39-10748407231171933] Liu W. Zhu J. Liu J. Guo Q . (2015). Psychological state and needs of family member caregivers for victims of traumatic brain injury: A cross-sectional descriptive study. International Journal of Nursing Sciences, 2(3), 231–236. 10.1016/j.ijnss.2015.07.001

[bibr40-10748407231171933] Lizarondo L. Stern C. Carrier J. Godfrey C. Rieger K. Salmond S. Apostolo J. Kirkpatrick P. Loveday H . (2020). Chapter 8: Mixed methods systematic reviews. In Aromataris E. JBI manual for evidence synthesis (pp. 270–307). JBI. 10.46658/JBIMES-20-09 32813460

[bibr41-10748407231171933] Maas A. I. Menon D. K. Adelson P. D. Andelic N. Bell M. J. Belli A. Bragge P. Brazinova A. Buki A. Chesnut R. M. Citerio G. Coburn M. Cooper D. J. Crowder A. T. Czeiter E. Czosnyka M. Diaz-Arrastia R. Derier J. P. Duhamie A. C. Yaff K . (2017). Traumatic brain injury: Integrated approaches to improve prevention, clinical care, and research. The Lancet Neurology, 16(12), 987–1048. 29122524 10.1016/S1474-4422(17)30371-X

[bibr42-10748407231171933] Man D. W. K. Lam C. S. Bard C. C . (2003). Development and application of the Family Empowerment Questionnaire in brain injury. Brain Injury, 17(5), 437–450. 10.1080/0269905031000070152 12745715

[bibr43-10748407231171933] Manskow U. S. Friborg O. Roe C. Braine M. Damsgard E. Anke A . (2017). Patterns of change and stability in caregiver burden and life satisfaction from 1 to 2 years after severe traumatic brain injury: A Norwegian longitudinal study. Neurorehabilitation, 40(2), 211–222. 10.3233/NRE-161406 27935561

[bibr44-10748407231171933] McIntyre M. Ehrlich C. Kendall E . (2020). Informal care management after traumatic brain injury: Perspectives on informal carer workload and capacity. Disability & Rehabilitation, 42(6), 754–762. 10.1080/09638288.2018.1508511 30326760

[bibr45-10748407231171933] Mehta P. Sharma K . (2014). Leadership: Determinant of women empowerment. SCMS Journal of Indian Management, 11(2), 5–10.

[bibr46-10748407231171933] Moola S. Munn Z. Tufanaru C. Aromataris E. Sears K. Sfetcu R. Currie M. Lisy K. Qureshi R. Mattis P. Mu P . (2020). Chapter 7: Systematic reviews of etiology and risk. In Aromataris E. (Eds.), JBI manual for evidence synthesis (pp. 217–267). JBI. 10.46658/JBIMES-20-08

[bibr47-10748407231171933] Nguyen R. Fiest K. M. McChesney J. Kwon C. S. Jette N. Frolkis A. D. Atta C. Mah S. Dhaliwal H. Reid A. Pringsheim T. Dykemann J. Gallagher C . (2016). The international incidence of traumatic brain injury: A systematic review and meta-analysis. Canadian Journal of Neurological Sciences, 43(6), 774–785. 10.1017/cjn.2016.290 27670907

[bibr48-10748407231171933] Norup A. Perrin P. B. Cuberos-Urbano G. Anke A. Andelic N. Doyle S. T. Cristina Quijano M. Caracuel A. Dulce M. Gudalupe Espinosa J. I. Carlos Arango-Lasprilla J . (2015). Family needs after brain injury: A cross cultural study. NeuroRehabilitation, 36(2), 203–214. 10.3233/NRE-151208 26410614

[bibr49-10748407231171933] Nygårdh A. Malm D. Wikby K. Ahlstrom G . (2012). Empowerment intervention in outpatient care of persons with chronic kidney disease pre-dialysis. Nephrology Nursing Journal, 39(4), 285–293. 23061113

[bibr50-10748407231171933] Oyesanya T . (2017). The experience of patients with ABI and their families during the hospital stay: A systematic review of qualitative literature. Brain Injury, 31(2), 151–173. 10.1080/02699052.2016.1225987 28055226 PMC5605764

[bibr51-10748407231171933] Page M. J. McKenzie J. E. Bossuyt P. M. Boutron I. Hoffmann T. C. Mulrow C. D. Shamseer L. Tetzlaff J. M. Akl E. A. Brennan S. E. Chou R. Glanville J. Grimshaw J. M. Hrobjartsson A. Lalu M. M. Tianjin L. Loder E. W. Mayo-Wilson E. . . .Moher D . (2021). The PRISMA 2020 statement: An updated guideline for reporting systematic reviews. International Journal of Surgery, 88, Article 105906. 10.1016/j.jclinepi.2021.03.001 33789826

[bibr52-10748407231171933] Papathanasiou I. V. Fradelos E. C. Kleisiaris C. F. Tsaras K. Kalota M. A. Kourkouta L . (2014). Motivation, leadership, empowerment and confidence: Their relation with nurses’ burnout. Materia Socio-medica, 26(6), 405–410. 10.5455/msm.2014.26.405-410 25685089 PMC4314154

[bibr53-10748407231171933] Rasmussen M. S. Arango-Lasprilla J. C. Andelic N. Nordenmark T. H. Soberg H. L . (2020). Mental health and family functioning in patients and their family members after traumatic brain injury: A cross-sectional study. Brain Sciences, 10(10), Article 670. 10.3390/brainsci10100670 PMC7600942 32992808

[bibr54-10748407231171933] Rodwell C. M . (1996). An analysis of the concept of empowerment. Journal of Advanced Nursing, 23(2), 305–313. 10.1111/j.1365-2648.1996.tb02672.x 8708244

[bibr55-10748407231171933] Rubin A. Babbie E. R . (2016). Empowerment series: Research methods for social work. Cengage Learning.

[bibr56-10748407231171933] Sakanashi S. Fujita K . (2017). Empowerment of family caregivers of adults and elderly persons: A concept analysis. International Journal of Nursing Practice, 23(5), Article e12573. 10.1111/ijn.12573 28691266

[bibr57-10748407231171933] Schutz R. E. Coats H. L. Engelberg R. A. Curtis J. R. Creutzfeldt C. J . (2017). Is there hope? Is she there? How families and clinicians experience severe acute brain injury. Journal of Palliative Medicine, 20(2), 170–176. 10.1089/jpm.2016.0286 27763820

[bibr58-10748407231171933] Sigurdardottir A. K. Leino-Kilpi H. Charalambous A. Katajisto J. Stark Å. J. Sourtzi P. Valkeapää K . (2015). Fulfilment of knowledge expectations among family members of patients undergoing arthroplasty: A European perspective. Scandinavian Journal of Caring Sciences, 29(4), 615–624. 10.1111/scs.12199 25648518

[bibr59-10748407231171933] Vaismoradi M. Turunen H. Bondas T . (2013). Content analysis and thematic analysis: Implications for conducting a qualitative descriptive study. Nursing & Health Sciences, 15(3), 398–405. 10.1111/nhs.12048 23480423

[bibr60-10748407231171933] Wåhlin I . (2017). Empowerment in critical care—A concept analysis. Scandinavian Journal of Caring Sciences, 31(1), 164–174. 10.1111/scs.12331 27164009

[bibr61-10748407231171933] Wetzig K. Mitchell M . (2017). The needs of families of ICU trauma patients: An integrative review. Intensive & Critical Care Nursing, 41, 63–70. 10.1016/j.iccn.2017.02.006 28366520

