# Supplementary material for: Empowering Support for Family Members of Brain Injury Patients in the Acute Phase of Hospital Care: A Mixed-Methods Systematic Review
Source: J Fam Nurs. 2023 May 16;30(1):50–67. doi: 10.1177/10748407231171933 (PMC10788044; doi:10.1177/10748407231171933)
Supplement: sj-pdf-2-jfn-10.1177_10748407231171933 – Supplemental material for Empowering Support for Family Members of Brain Injury Patients in the Acute Phase of Hospital Care: A Mixed-Methods Systematic Review [file sj-pdf-2-jfn-10.1177_10748407231171933.pdf]

Supplementary file 2. Description of the phases of the analysis process

**Main theme**	**TBI Patients' Family Members Empowering Support in Acute Care Hospitals**		
**Theme**	Needs-based informational support to empower the family members		
**Sub theme**	Information about TBI patients' health conditions in the acute phase	Trustworthy and adequate information about the progress of the TBI patient's care	Practical information about the uncertain future
**Codes example**	• needs for early diagnosis of the patient's brain injury<br>• meaning of traumatic brain injury<br>• needs for physical and cognitive symptoms of TBI causes<br>• changes and progress in the patient's health conditions<br>• predictable results in the patient's health conditions	• FMs felt it was important that the healthcare professionals ensured the understanding of the received information<br>• the information must be honest, consistent, up to date, concrete and regular<br>• timely information increased FMs' understanding of brain injury and long-term prognosis<br>• information wanted to be in oral and written<br>• FMs' guidance and information in a calm environment	• the needs of FMs focused on planning for the future<br>• worry about how TBI will affect the family life<br>• TBI consequences on the working life of a person with TBI<br>• lack of knowledge about the future reduced the feeling of FMs that they were ready to manage TBI persons care independently at home.
**Studies**	Adams & Dahdah, 2016; Arango-Lasprilla et al., 2010; Bellon et al., 2015; Calvete & Arroyabe, 2012; Choustikova et al., 2020; Degeneffe & Bursnall, 2015; Doyle et al., 2013; Gan et al., 2010; Kanmani & Raju, 2019; Keenan & Joseph, 2010; Kanmani & Raju, 2019; Kreitzer et al., 2019; Lefebvre & Levert, 2012a; Liu et al., 2015	Gan et al., 2010; de Goumoëns et al., 2019; Keenan & Joseph, 2010; Kreitzer et al., 2019; Lefebvre & Levert, 2012b	Abrahamson et al., 2017; Adams & Dahdah, 2016; Arango-Lasprilla et al., 2010; Calvete & Arroyabe, 2012; Choustikova et al., 2020; Degeneffe & Bursnall, 2015; Dillahunt-Aspillaga et al., 2013; Doyle et al., 2013; Gan et al., 2010; Keenan & Joseph, 2010; Kreitzer et al., 2019; Lefebvre & Levert, 2012a

Supplementary file 2. Description of the phases of the analysis process

**Main theme**	**TBI Patients' Family Members Empowering Support in Acute Care Hospitals**	
**Theme**	Participatory support to empower the family members	
**Sub theme**	Participating in the TBI patient's care	Family members' involvement in the TBI patient's transfers and discharge plans
**Codes example**	• the presence of a FMs next to the TBI patient was felt to be the primary way to participate in the patient's care in the acute phases of treatment.<br>• be part of the TBI patients' treatment process<br>• participate concretely in the patient's care<br>• being by the patient's side increased the optimistic feeling of the FMs that the TBI patient's recovery is progressing recover<br>• FMs need to feel useful and involved in the patient's care	• FMs need to be involved in the patient's treatment plan and discharge plan<br>• involving FMs in the discharge plan increased the commitment and preparedness of FMs to caring for the patient<br>• waiting, lack of knowing and uncertainty about transfers and discharge plans increased a feeling of helplessness and worry in FMs<br>• involving FMs in the planning of discharge made it possible to avoid the hospital cycle
**Studies**	Bellon et al., 2015; Calvete & Arroyabe, 2012; Degeneffe & Bursnall, 2015; de Goumoëns et al., 2019; Kanmani & Raju, 2019; Keenan & Joseph, 2010; Lefebvre & Levert, 2012a	Abrahamson et al., 2017; Calvete & Arroyabe, 2012; Keenan & Joseph, 2010; Kreitzer et al., 2019;Lefebvre & Levert, 2012a; Liu et al., 2015; Norup et al., 2015

Supplementary file 2. Description of the phases of the analysis process

**Main theme**	**TBI Patients' Family Members Empowering Support in Acute Care Hospitals**		
**Theme**	Competent and interprofessional support to empower the family members		
**Sub theme**	Confidence in the competence of healthcare professionals	Maintaining a sense of hope	Interprofessional collaboration in supporting family members
**Codes example**	• the support of professionals in the acute phase of the TBI patient's treatment was primary and important for FMs<br>• competence was one of the key factors that influenced the feeling of empowerment of FMs from the support received from professionals.<br>• respectful, reliable, communicative, listening, and empathetic relationship<br>• competence of the healthcare professionals affected both the survival and coping of FMs<br>• no one of healthcare professionals inquired about the well-being of FMs	• it was very important for FMs to feel hope for the patient's treatment<br>• FMs wanted healthcare professionals to give them hope for the future<br>• hope increased the feeling of empowerment of FMs<br>• the need for hope of FMs remained throughout the patient's treatment	• healthcare professionals should ensure that the FMs received the support and assistance they need from the right professionals.<br>• FMs need specialists in different fields to help them cope with a new life situation<br>• interprofessional support reduced FMs’ stress<br>• cooperation improved FMs’ well-being
**Studies**	Abrahamson et al., 2017; Calvete & Arroyabe, 2012; Choustikova et al., 2020; Gan et al., 2010; de Goumoëns et al., 2019; Holloway et al., 2019; Keenan & Joseph, 2010; Kreitzer et al., 2019; Lefebvre & Levert, 2012b; Liu et al., 2015	Arango-Lasprilla et al., 2010; Bellon et al., 2015; Calvete & Arroyabe, 2012; Keenan & Joseph, 2010, Lefebvre & Levert, 2012a; Liu et al., 2015; Schutz et al., 2017	Abrahamson et al., 2017; Choustikova et al., 2020; Gan et al., 2010; Norup et al., 2015; Keenan & Joseph, 2010; Lefebvre & Levert, 2012b; Liu et al., 2015

Supplementary file 2. Description of the phases of the analysis process

**Main theme**	**TBI Patients' Family Members Empowering Support in Acute Care Hospitals**		
**Theme**	Community support to empower the family members		
**Sub theme**	A good social support network	Information about different peer support services	Ensuring continuity of care after the TBI patient's hospital
**Codes example**	• it was important to get support from other family members and friends<br>• considering the effect of community support on the fatigue and burden of FMs<br>• support from outside the hospital was important<br>• community support became more important towards the end of the patient's acute care when the needs of FMs focused more on life management and on combining patient care and family's everyday life.	• in the early phases of TBI patients' treatment, it was important to have peer support, a person with whom you can share your feelings and experiences<br>• the stories and experiences of peers also gave the families a sense of hope for their loved one's recovery<br>• peer support services offered timely and appropriate crisis support for FMs<br>• information about peer support services was often given too late	• someone who takes care of and acts as a link in organizing the patient's discharge and services<br>• the contact information of the person who can be contacted after returning home<br>• the coordination of care and support services help FMs to operate in a complex health service system<br>• FMs hoped for continuity between hospitals, support services, and long-term care services
**Studies**	Calvete & Arroyabe, 2012; Holloway et al., 2019; Keenan & Joseph, 2010; Lefebvre & Levert, 2012a	Adams & Dahdah, 2016; Arango-Lasprilla et al., 2010; Bellon et al., 2015; Gan et al., 2010; Keenan & Joseph, 2010; Norup et al., 2015	Abrahamson et al., 2017; Bellon et al., 2015; Doyle et al., 2013; Holloway et al., 2019; Norup et al., 2015; Lefebvre & Levert, 2012a; Liu et al., 2015
